# A lithotrophic microbial fuel cell operated with pseudomonads-dominated iron-oxidizing bacteria enriched at the anode

**DOI:** 10.1111/1751-7915.12267

**Published:** 2015-02-25

**Authors:** Thuy Thu Nguyen, Tha Thanh Thi Luong, Phuong Hoang Nguyen Tran, Ha Thi Viet Bui, Huy Quang Nguyen, Hang Thuy Dinh, Byung Hong Kim, Hai The Pham

**Affiliations:** 1 Research group for Physiology and Applications of Microorganisms (PHAM group) at Center for Life Science Research, Vietnam National University – University of ScienceNguyen Trai 334, Thanh Xuan, Hanoi, Vietnam; 2 Department of Microbiology, Faculty of Biology, Vietnam National University – University of ScienceNguyen Trai 334, Thanh Xuan, Hanoi, Vietnam; 3 Department of Biochemistry, Faculty of Biology, Vietnam National University – University of ScienceNguyen Trai 334, Thanh Xuan, Hanoi, Vietnam; 4 Laboratory of Microbial Ecology, Institute of Microbiology and Biology, Vietnam National UniversityXuan Thuy 144, Cau Giay, Hanoi, Vietnam; 5 Korea Institute of Science and TechnologyHwarangno 14-gil, 5 Seongbuk-gu, Seoul, 136-791, Korea; 6 Fuel Cell Institute, National University of MalaysiaBangi, 43600 UKM, Selangor, Malaysia; 7 School of Municipal and Environmental Engineering, Harbin Institute of Technology73 Huanghe Road, Nangang District, Harbin, 150090, China

## Abstract

In this study, we attempted to enrich neutrophilic iron bacteria in a microbial fuel cell (MFC)-type reactor in order to develop a lithotrophic MFC system that can utilize ferrous iron as an inorganic electron donor and operate at neutral pHs. Electrical currents were steadily generated at an average level of 0.6 mA (or 0.024 mA cm^–2^ of membrane area) in reactors initially inoculated with microbial sources and operated with 20 mM Fe^2+^ as the sole electron donor and 10 ohm external resistance; whereas in an uninoculated reactor (the control), the average current level only reached 0.2 mA (or 0.008 mA cm^–2^ of membrane area). In an inoculated MFC, the generation of electrical currents was correlated with increases in cell density of bacteria in the anode suspension and coupled with the oxidation of ferrous iron. Cultivation-based and denaturing gradient gel electrophoresis analyses both show the dominance of some *Pseudomonas* species in the anode communities of the MFCs. Fluorescent in-situ hybridization results revealed significant increases of neutrophilic iron-oxidizing bacteria in the anode community of an inoculated MFC. The results, altogether, prove the successful development of a lithotrophic MFC system with iron bacteria enriched at its anode and suggest a chemolithotrophic anode reaction involving some *Pseudomonas* species as key players in such a system. The system potentially offers unique applications, such as accelerated bioremediation or on-site biodetection of iron and/or manganese in water samples.

## Introduction

The research interest in microbial fuel cells (MFCs) has increased recently, due to their unique property of exploiting microbial activity to generate electricity from energy-storing substances. In MFCs, microorganisms act as biocatalysts to convert chemical energy comprised in electron donors to electrical energy (Allen and Bennetto, 1993; Logan *et al*., 2006). These systems can also be modified (and assisted with energy) to become microbial electrolysis cells, in which hydrogen or other substances can be produced (Logan *et al*., 2006; Rozendal *et al*., 2006; Rabaey and Rozendal, 2010). ‘Bioelectrochemical systems’ is therefore a broad sense term to designate all kinds of these systems (Rabaey *et al*., 2007).

Up to now, the majority of MFC researches have been focused on optimization of the device for the recovery of energy from biomass (mostly in waste) or from light or for bioremediation or the production of future clean energy (Rosenbaum *et al*., 2010; Lovley and Nevin, 2011; Wang and Ren, 2013). However, due to some performance-limiting factors, including the microbial activity, the electron transfer process, the internal resistance of the device and particularly the cathode reaction rate (Pham *et al*., 2006; Kim *et al*., 2007), the maximum power output (per volume unit) of an MFC is still limited. Moreover, scale-up difficulties also hindered the realization of this technology in the field of energy recovery (Rozendal *et al*., 2008; Cheng *et al*., 2014). Therefore, currently, many MFC researches are being directed towards exploiting the special characteristics of MFCs for environmental bioremediation or for biosynthesis and the development of biosensors (Kim *et al*., 2007; Rabaey and Rozendal, 2010; Lovley and Nevin, 2011; Arends and Verstraete, 2012).

There have been MFCs utilizing various types of substrates, including a wide range of soluble or dissolved complex organic matter (Pant *et al*., 2010). Most substrates tested in MFCs are indeed artificial or real wastewaters containing different kinds of compounds (Rabaey *et al*., 2007; Pant *et al*., 2010). There are also MFCs operated with single substances such as acetate, formate or Acid orange 7, etc. (Lee *et al*., 2003; Ha *et al*., 2008; Fernando *et al*., 2014). It is common that most substrates (electron donors) used in MFC systems so far are organic (Pant *et al*., 2010), meaning that bacteria in these systems are heterotrophic. There have been only few reports about the use of an inorganic electron donor, such as sulfide, in a MFC (Rabaey *et al*., 2006) but as for sulfide, it can only be used in the presence of another organic electron donor, i.e. acetate. Little is known about whether an ‘inorganic’ MFC that utilizes metal ions can actually function.

In principle, the development of such an ‘inorganic’ MFC operated with metal ions such as ferrous ions should be feasible because there exist a group of bacteria that can oxidize ferrous ions to gain energy – the chemolithotrophic iron-oxidizing bacteria (or iron bacteria) (Cullimore and McCann, 1978; Hedrich *et al*., 2011). Taxonomically, these bacteria are classified into several groups but most of them belong to proteobacteria (Hedrich *et al*., 2011). The acidophilic iron-oxidizing proteobacteria, such as *Acidithiobacillus ferrooxidans*, were probably considered typical iron bacteria (Hedrich *et al*., 2011). However, there are also phototrophic iron bacteria, neutrophilic iron bacteria that respire on nitrate or even neutrophilic aerobic iron bacteria, including some *Pseudomonas* species (Sudek *et al*., 2009; Hedrich *et al*., 2011).

In this study, we attempted to enrich neutrophilic iron bacteria in a MFC in order to develop an iron-oxidizing MFC system that can operate at neutral pHs, as this condition will be convenient for practical applications. Such a lithotrophic MFC can be not only scientifically interesting but also promisingly used as a biosensor detecting iron or as a bioremediation means to remove iron or other metal pollutants from water.

## Results

### Generation of electrical currents in MFCs fed with ferrous iron as the sole electron donor – an indication of the enrichment of iron-oxidizing bacteria

**Fig 1:**
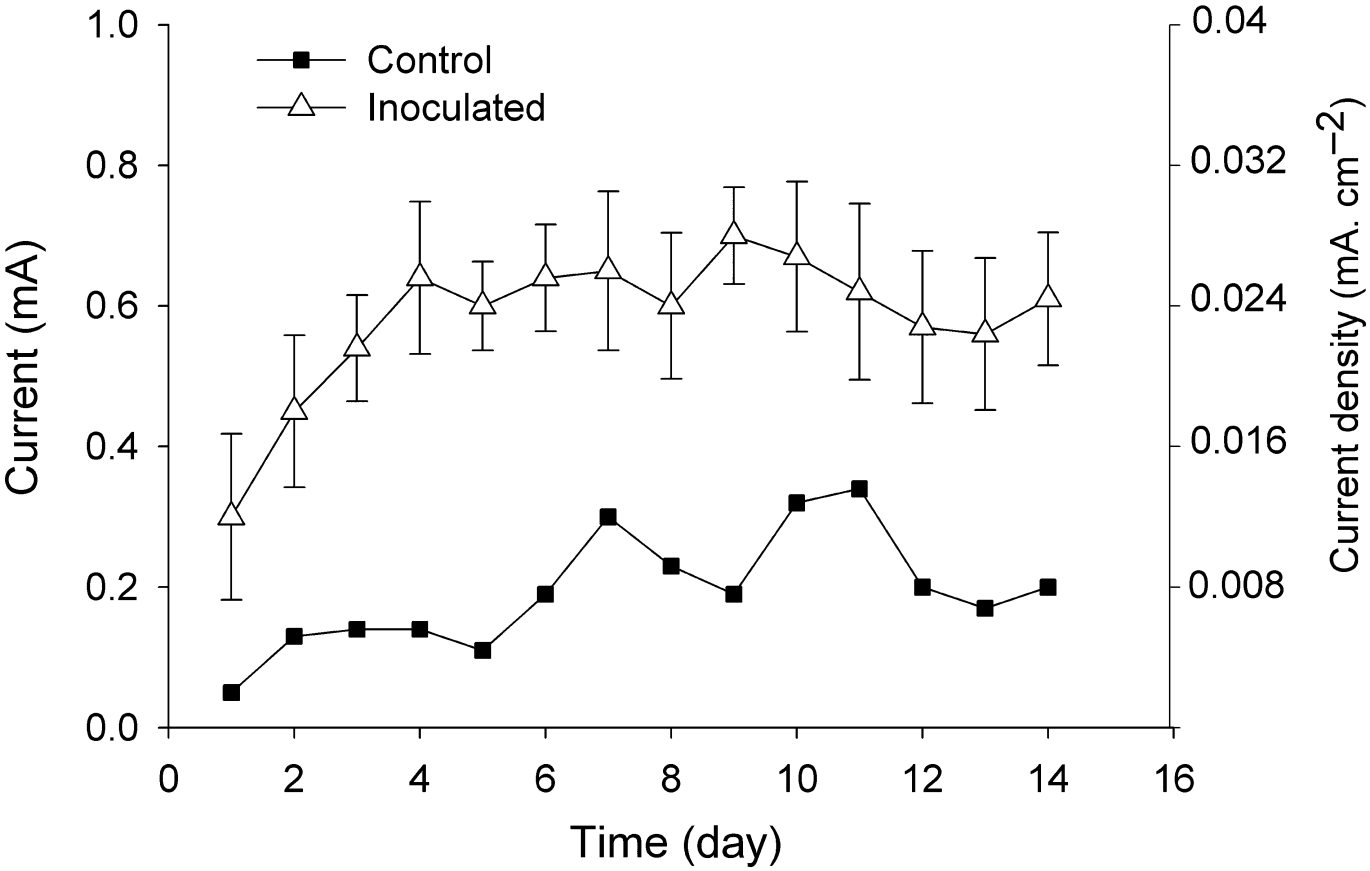
Typical patterns of the generations of electrical currents by an inoculated MFC and the control (uninoculated but not abiotic) during the enrichment period. The MFCs were operated with ferrous iron as the only electron donor in the anodes (see *Experimental procedures*) and with a 10 ohm external resistor, at 25°C. Each inoculated MFC was inoculated with the mud from a natural stream suspected to contain iron bacteria. The control was not inoculated with any microbial source at the beginning. Each data point is an average current per batch on the corresponding day, generated by the corresponding MFC(s).

Several modified National Centre for Biotechnology Education (UK) (NCBE)-type MFC reactors were set up, inoculated and operated with a modified M9 medium containing only Fe^2+^ (20 mM) as the sole electron donor at the anode. Within the first 2 days of operation, all of the reactors already began to generate electrical currents (Fig. 1). After 2 weeks of operation, the electrical currents of the reactors were steady. The generation of electrical currents while being fed with Fe^2+^ as the only electron donor is the first evidence of the function of the reactors as MFCs and of the enrichment of iron-oxidizing bacteria.

Differences in the levels of current generation could be clearly observed (*P* < 0.05) between an MFC that was initially inoculated with a microbial source (an inoculated MFC) and a MFC that was not (the control). At the steady state, with 20 mM Fe^2+^ supplied into the anode compartment, the average currents of an inoculated MFC was 0.6 ± 0.11 mA (equivalent to 0.024 ± 0.0044 mA cm^−2^ membrane area) while that of the control was only 0.2 mA (equivalent to 0.008 mA cm^−2^ membrane area) (Fig. 1). The amount of coulombs produced by the former was even six times as much as that produced by the latter (Fig. S2). The differences of inoculated MFCs versus the control imply that the generation of current in an inoculated MFC is due to electroactive bacteria that might be feasibly enriched from the initial microbial source. Such a source probably allows the selection from a large microbial community for electrochemically active bacteria that can use Fe^2+^ as the electron donor. Thus in the control, without an initial inoculum, such bacteria might not be enriched. The current generated by the control, although at low levels, might be due partially to plain chemical reactions (as described later) and partially to the activity of contaminating bacteria from the surroundings. These bacteria might gradually adapt to the anode conditions but their electrochemical activity might not be competent enough.

### The generation of electricity in relation to the microbial activity in the MFCs

**Fig 2:**
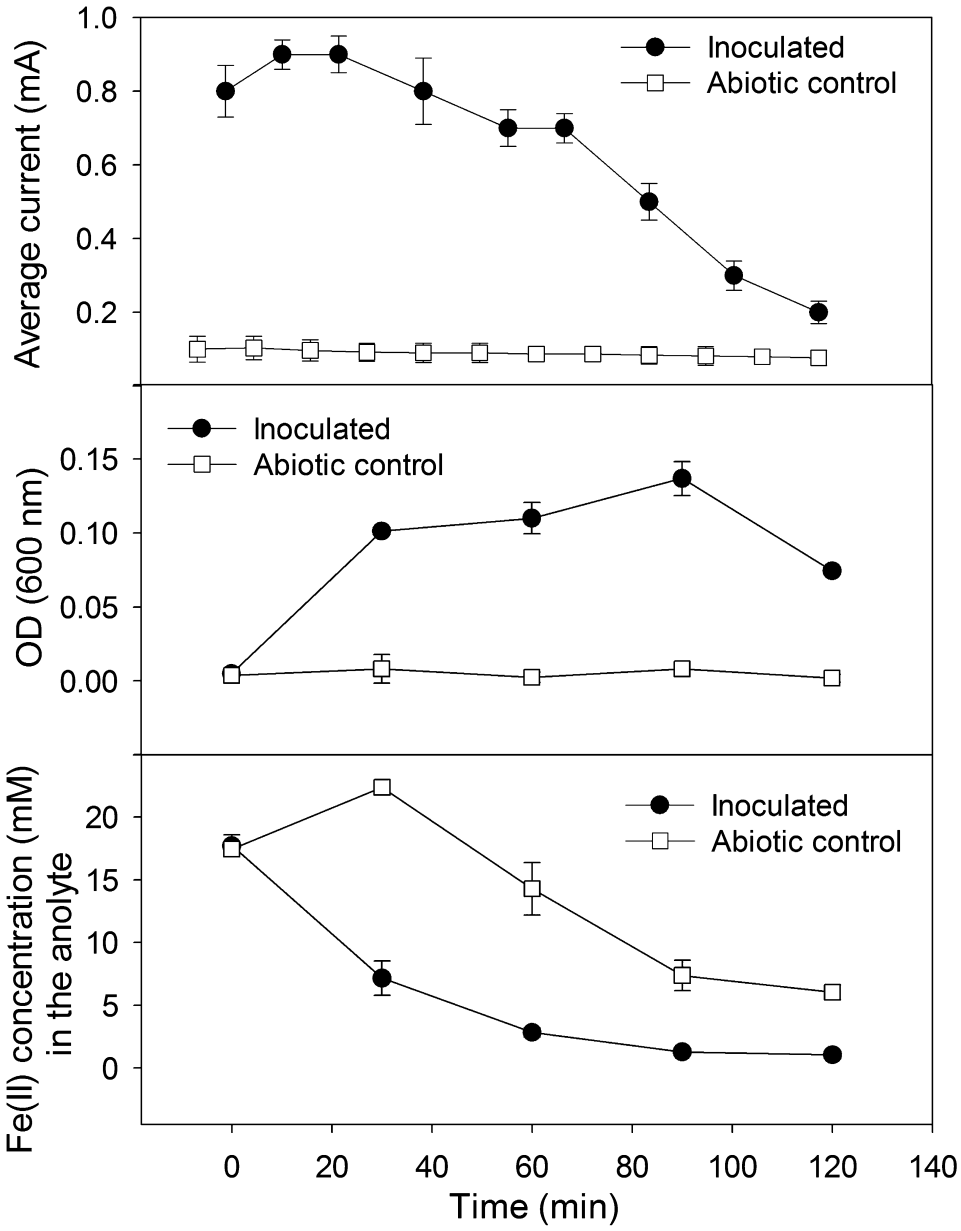
Typical patterns of changes of the generated current (top), the optical density at the wavelength of 600 nm of the anode suspension (center), and the concentration of ferrous iron in the anolytes (bottom) during an operational batch of a MFC inoculated with bacteria in comparison with those of the abiotic control. The MFCs were operated with a 10 ohm external resistor at 25°C. Error bars represent standard deviations.

To prove that the generation of electricity in the MFCs was not due to plain chemical reactions, an abiotic control with the anode compartment sterilized (see *Experimental procedures*) was tested with Fe^2+^. Under such conditions [with optical density (OD) (600 nm) values being approximately 0 – Fig. 2 ], when only abiotically chemical reactions could occur, the current generated in an operational batch was very limited (0.1 ± 0.02 mA) and distinctively much lower than that of an inoculated MFC (under biotic conditions) (Fig. 2). Noticeably, the current generated by an abiotic control, although reaching certain levels right after the supply of Fe^2+^, rapidly decreased down to near the bottom level during a batch. Together with a generated current that remained at significant levels for a long time in an inoculated MFC, increases in the number of bacterial cells [reflected by OD (600 nm) values] were also observed at some points (Fig. 2). However, it seems that under both abiotic and biotic conditions, the trends of change of the Fe(II) concentration were similar (Fig 2). These results proved that abiotically chemical oxidation of ferrous ions does occur in the anode of our reactors, but the electrode itself was probably not an electron acceptor for this abiotic oxidation, resulting in little electricity generated by the abiotic control. Probably, ferrous ions were abiotically oxidized by oxygen diffused from the cathode through the membrane, as shown elsewhere (Pham *et al*., 2004). Only in a MFC inoculated with bacteria, a significant electrical current could be generated (0.6 ± 0.11 mA versus 0.1 ± 0.02 mA of the abiotic control). It could be the result of efficient interactions of the enriched bacteria with the anodic electrode (the microbial activity) – a property that the abiotic control does not have. This also implies that the consortium of bacteria that was enriched in an inoculated MFC can oxidize Fe^2+^ before transferring electrons to the electrode. Ferric precipitate was also observed more in the anode compartment of an inoculated MFC than in that of the abiotic control.

All the results reported above suggest that our inoculated MFCs were successfully developed and functioned as MFCs that generate electricity upon oxidizing ferrous ion, due to the electrochemical activity of the microbes enriched at the anodes.

### Culturable bacteria in the anode of iron-oxidizing MFCs

**Fig 3:**
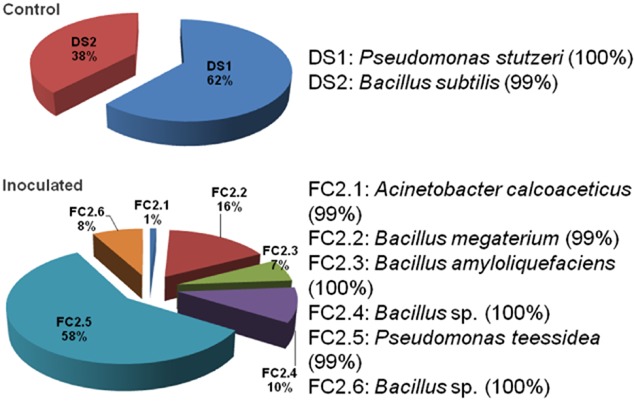
The levels of presence frequence (expressed as percentage per total culturable colonies) of the bacterial isolates from an inoculated MFC and the control. Isolation was done on solid Winogradsky medium. Isolates named with ‘DS’ were from the control, while those named with ‘FC2’ were from an inoculated MFC. Notes on the right indicate the proposed taxonomic identification of the corresponding isolates based on observations of their cell and colony morphology and analyses of their 16S rDNA sequences. The percentage of similarity between the 16S rDNA sequence of an isolate and that of the proposed species was shown in the brackets next to the corresponding note.

Culturable bacteria in the anode suspensions of an inoculated MFC and the control (that was not uninoculated) were grown and isolated on Winogradsky medium to find potential iron bacteria (Starosvetsky *et al*., 2008). The number and types of strains isolated from the inoculated MFC were different from those from the control (Fig. 3). Only two isolates could be cultivated from the anode of the control while six isolates could be obtained from the anode of the inoculated MFC. Colony plating and counting results showed the high presence frequence of isolate FC 2.5 in the microbial community of the studied inoculated MFC (Fig. 3). Results of 16S rDNA sequence analyses showed that strain FC 2.5 was close to *Pseudomonas teessidea* (99% sequence homology). Also based on 16S rDNA sequence analyses, some other strains could be identical to *Bacillus* sp. (FC 2.3 and FC 2.6) and other species such as *Acinetobacter* sp. (FC 2.1). These results are interesting because none of those isolates are related to popularly-known iron bacteria.

### Anode bacterial communities of iron-oxidizing MFCs

In order to analyse the bacterial community at the anode of each MFC, total DNA of bacterial cells in the anode suspension was extracted, together with total DNA of those scraped off from the electrode surface. Surprisingly, in all cases, very little DNA was obtained from the electrode surfaces (data not shown), indicating that bacteria did not occupy the anode surfaces. This result is also consistent with the fluorescent in situ hybridization (FISH) results reported later (Fig. 5).

**Fig 4:**
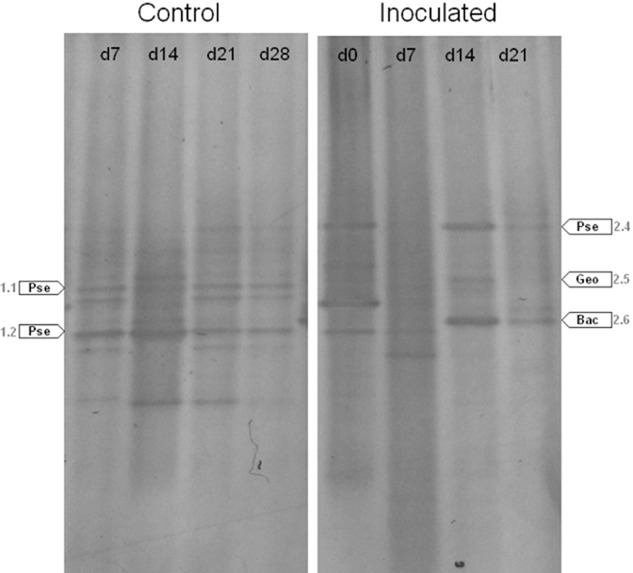
DGGE analysis of the anodic bacterial communities of the MFCs at different time points during the enrichment period. The note on each lane of a gel indicates the moment the sample was taken (for example, d7 = at the 7th day). A sample at d0 indeed resembles the corresponding inoculum. The note on each white arrow indicates the genus, 16S rDNA sequence of which has the highest similarity (> 98%) to the DNA sequence of the corresponding band on the gel (based on BLAST analysis). Pse = *Pseudomonas* sp., Geo = *Geobacter* sp., Bac = *Bacillus* sp. (The note next to each arrow is the assigned number of the corresponding band). The DGGE was repeated three times with three replicates of each sample. As the results of these repetitions were absolutely similar, only typical patterns were shown here.

Since no DNA was obtained from the electrode surfaces, only the bacterial compositions of the anode suspensions of the MFCs were analysed and compared by denaturing gradient gel electrophoresis (DGGE) (Fig. 4). DGGE patterns clearly showed that the anode communities enriched with and without a microbial source are significantly different and change with time in different manners. As can be seen in Fig. 4, in the control, there was a community that was established and became stable already after the first week of operation (with a rate of change of ∼ 0% week^−1^). That community appeared to consist of a number of species but no species seemed to dominate. Band sequence analyses showed that the presence of many *Pseudomonas* species was evident. In an inoculated MFC, in contrast, the microbial community still changed after the first week of operation (with a rate of change of ∼ 73 ± 12% week^−1^) and only showed a steady state after 2 weeks (still with a rate of change of ∼ 20 ± 10% week^−1^). Moreover, there appeared some species that dominate the community. Based on band sequence analyses, these species were suspected to be *Pseudomonas* sp., *Geobacter* sp. and *Bacillus* sp. (Fig. 4).

**Fig 5:**
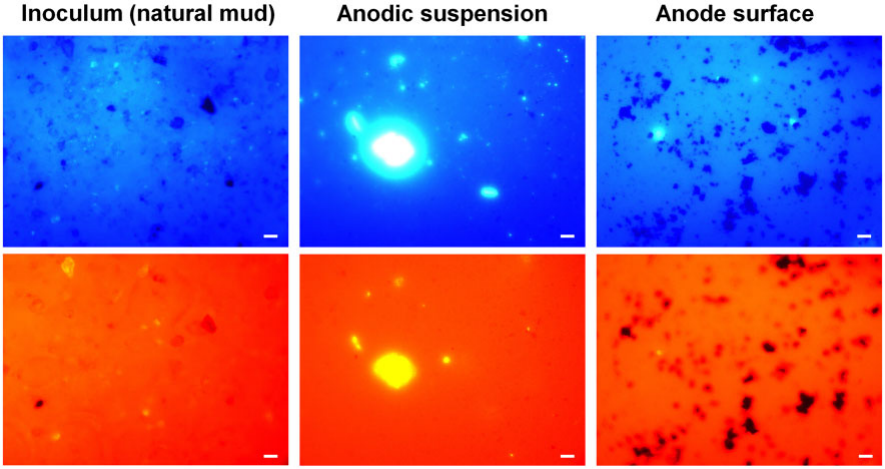
FISH analyses of iron bacteria in the inoculum and in the anode suspension as well as on the anode surface of an inoculated MFC. Upper are images created from fluorescent signals of DAPI, with white dots showing the presence of all bacteria in the samples. Lower are images created from fluorescent signals of Cy3 attached to the probe PS1, with white dots showing the presence of iron bacteria in the samples. Bars, 5 μm.

In order to assess the presence of iron bacteria, particularly neutrophilic iron-oxidizing ones, in the anode of an iron-oxidizing MFC, FISH analyses of anode surface samples and anode suspension samples of another inoculated MFC were carried out using probe PS 1. FISH images (Fig. 5) showed a significant level of PS 1 signal (white dots) reflecting the presence of iron bacteria in the anode suspension of that inoculated MFC. Analyses of these images by Image J revealed that the proportion of iron bacteria in the anode suspension of that MFC was 36.5% of the total bacteria, while that in the inoculum was only 25%. This indicates a significant increase in the quantity of iron bacteria in the anode suspension after the enrichment period. On the other hand, it is interesting to note that few iron bacteria, even few bacteria, were present on the anode surface. These results suggest that iron bacteria are mostly present and active in the anode suspension but not on the electrode surface.

## Discussion

### The successful development of iron-oxidizing MFCs

In this study, MFCs that can generate electricity upon utilizing ferrous iron as the electron donor have been experimented and shown to function. As mentioned, our results show that with a proper inoculum (i.e. collected from a site with a high chance to contain iron bacteria), a bacterial community can be enriched in the anode of such a reactor and responsible for the generation of electricity coupled to the oxidation of Fe^2+^. Indications of the successful enrichment of such a consortium include: (i) enrichment period current patterns that resemble those in other types of MFCs, (ii) the significant generation of electricity only when bacteria are present (Fig. 2) (*P* < 0.05) and (iii) the detection of bacterial communities including iron bacteria based on DGGE and FISH analyses.

Normally, the required time for an electrochemically active community to establish and stabilize is around 2 weeks (Kim *et al*., 2003; Rabaey and Verstraete, 2005) although shorter enrichment time (e.g. 5 days) has been reported (Ishii *et al*., 2014). The slow growth of iron bacteria at neutral pHs due to the lower Fe^2+^/Fe^3+^ redox potential (Hedrich *et al*., 2011) might be also an explanation for this relatively long enrichment time. The pattern of current generation of an inoculated MFC in this study in the first 2 weeks of operation is similar to that during the enrichment period of a typical MFC (Kim *et al*., 2004; Rabaey and Verstraete, 2005). During enrichment, shifts in the composition of the anode community (of an inoculated MFC) (Fig. 5) indicate a selection process in which only bacteria that can well adapt to the anode conditions (by being able to utilize Fe^2+^ and interact with the electrode) become dominant. This was also supported by the increased quantity of iron bacteria after enrichment, as demonstrated by the FISH results (Fig. 4). Similar community shifts during enrichment to finally shape a working electroactive community were also observed in other MFC systems (Aelterman *et al*., 2006; Pham *et al*., 2009). A noticeable point about our MFCs is that unlike other systems, they are not operated with organic matter as fuel, and thus require an anode community that contains not only electrochemically active bacteria but also chemolithotrophic ones living on ferrous ions. Our study demonstrates that the development of such an iron-oxidizing MFC system is feasible. There have been reports on MFC systems using iron-containing compounds or ferric iron reducing bacteria or even ferrous iron oxidizing ones at the cathodes (ter Heijne *et al*., 2006; 2007) but there has been no similar study on MFCs utilizing ferrous iron as the fuel.

### The role of the microbial source for inoculation

It is clearly shown in this study that an initial microbial source is essential for the establishment of a final working community that oxidizes Fe^2+^ and transfer electrons to the anode. Moreover, it appears that a natural source might enable the enrichment of a working community that is stable and performs well. The enrichment and stabilization of a working community from a natural microbial source may take longer but this is definitely not a critical matter. Indeed, most well-performing and stable open-system MFCs are operated with mixed cultures, enriched from natural microbial sources (Kim *et al*., 2004; Logan and Regan, 2006). The fact that an iron-oxidizing MFC can function well with an anode consortium enriched from a natural source also means that practical development of this type of MFC is straightforward.

### The correlation between the composition of an anode community and its performance

The differences in the composition between the microbial communities, as shown by both solid-medium growth and DGGE results, might account for the differences in their performance. The bacterial community of an inoculated MFC is different from that of the control (the uninoculated but not abiotic), particularly in the aspect that the former is dominated by some species while the latter is not. This definitely has some links with the differences in their performance: the former performs distinctively better than the latter. The correlation between the composition of an anode community and its performance has been reported previously (Pham *et al*., 2008a; 2009).

It should be also noted from both the solid medium growth results and the DGGE results that the number of species in the anode community of each MFC is small (less than 10), i.e. the community is basically not very diverse. This could be explained by the poor nutrient conditions at the anode of each MFC, forcing the community to carry out chemoautotrophic or chemolithotrophy-associated metabolism to survive. Such conditions definitely select for a community so specialized to adapt that it contains only a limited number of species. Indeed, unique communities were observed in MFC systems operated with specific substrates such as formate or acetate (Lee *et al*., 2003; Ha *et al*., 2008).

### Hypothesis about the electron transfer mechanism in an iron-oxidizing MFC

**Fig 6:**
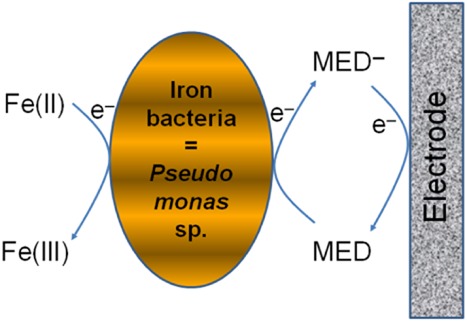
The hypothesized mechanism of electron transfer occurring at the anode of the iron-oxidizing MFC in this study. MED: mediator.

Our results, altogether, reveal several striking facts. First, both solid-medium growth results and DGGE results indicate the presence of *Pseudomonas* species in the anode communities of all the MFCs and their dominance in an inoculated MFC, which is a well-performing MFC. Second, bacteria detected by probe PS 1, supposed to be neutrophilic iron bacteria, became increased in quantity in the anode community of a well-performing MFC, as shown by FISH results. Third, bacteria, including iron bacteria, could be found in the anode suspensions of the MFCs but hardly detected on the anode surfaces. These facts lead us to a hypothesis that iron-oxidizing bacteria are present in the anode communities of our iron-oxidizing MFCs but they do not directly transfer electrons to the electrode. In the studies on direct electron transfer to electrodes (either via outer membrane proteins or nanowires), bacteria could always be observed on the electrode surfaces (Kim *et al*., 1999; Reguera *et al*., 2005). Only bacteria that can indirectly transfer electrons via chemicals or self-produced mediators are present and active in the anode suspension (Allen and Bennetto, 1993; Rabaey *et al*., 2005; Pham *et al*., 2008b). *Pseudomonas* species have been well known as electrochemically active heterotrophic bacteria that can self-produce electron mediators to reduce an electrode upon oxidizing organic matter (Rabaey *et al*., 2004; 2005). However, some *Pseudomonas* species have been reported to be able to chemolithotrophically metabolize Fe^2+^ at neutral pHs (Straub *et al*., 1996; Sudek *et al*., 2009). Considering those facts and the fact that *Pseudomonas* species are dominant in the anode of all the MFCs, our hypothesis for the anode electron transfer in our iron-oxidizing MFC is as follows (Fig. 6): Some *Pseudomonas* species could be actually the dominant ‘neutrophilic iron bacteria’ that can oxidize Fe^2+^ and transfer electrons to the anodic electrode via their self-produced mediators. In an inoculated MFC, the enriched anode consortium might contain these specialized *Pseudomonas* species that dominate and enable the MFC to function well, generating remarkable currents. In the control (uninoculated but not abiotic), probably some *Pseudomonas* cells that somewhat can do the same ‘job’ from surroundings could invade the anode but their activities might not be specific or efficient enough to enable a significant generation of electricity.

If our hypothesis is true, it is probable that the probe PS 1 used in the FISH experiments might unspecifically hybridize DNAs or RNAs of *Pseudomonas* species and thus could also detect *Pseudomonas* species instead of the targeted ‘neutrophilic iron bacteria’. PS1 was, in fact, designed upon aligning the 16S rDNA sequences of various groups of neutrophilic iron bacteria, particularly those of *Leptothrix* group (Siering and Ghiorse, 1997). However, it was not certain that this probe did not give positive results when tested with *Pseudomonas* species.

The presence of *Geobacter* sp. in the anode community of an inoculated MFC is interesting yet remains questioned because several *Geobacter* sp. are known electroactive bacteria that transfer electrons to the electrode by means of direct contacts (e.g. conductive pili) (Lovley and Nevin, 2011) while few bacteria were found on the anode surface of our inoculated MFC. The presence of *Bacillus* sp., which are heterotrophic Gram-positive bacteria that have not been shown to have electrochemical activities, is not either interpretable. Probably, in our iron-oxidizing MFCs, *Geobacter* and *Bacillus* species only act as opportunistic bacteria that take advantage of the output from iron oxidation autotrophy.

### Potential applications of iron-oxidizing MFCs in this study

It is noticeable that with the anode community dominated by neutrophilic iron-oxidizing bacteria, most possibly *Pseudomonas* species, our MFC system can be operated at pHs around 7. This enables a convenient operation and handling of the MFC. These MFC systems could be used for an accelerated bioremediation of iron in water samples, as the soluble ferrous salts could be converted to insoluble ferric salts by the activity of the anode electroactive iron-oxidizing community. They can also be potentially used as on-site biosensors to detect and monitor iron or manganese (as iron bacteria also oxidize Mn) in water samples, because of the correlation between their generated current and the concentration of Fe^2+^. This would be a substantial environmental application, if one considers the use of such an on-site biosensor in remote areas (in Vietnam for instance) where ground water is used as the major water source.

Overall, the results of this research showed that it is feasible to develop a lithotrophic MFC with an anode enriched with an iron-oxidizing electroactive bacterial consortium that can function well at neutral pHs. The key role of some *Pseudomonas* species as lithotrophic neutrophilic iron bacteria is probably the most convincing explanation for the anode electron transfer in such a MFC system. The MFC, with its unique properties, would offer potential applications in bioremediation or biomonitoring of iron in water. However, further studies are required to realize these potentials.

## Experimental procedures

### Fabrication and operation of MFC reactors

The MFC reactors in this study were fabricated following the NCBE model (Allen and Bennetto, 1993) with some modifications (Fig. S1). Each reactor consisted of two big poly-acrylic frames (12 cm × 12 cm × 2 cm) and two small poly-acrylic rectangle-holed frames of anode and cathode compartments (8 cm × 8 cm × 1.5 cm). The dimension of each rectangle hole on each small frame was 5 cm × 5 cm and thus each compartment had the dimension of 5 cm × 5 cm × 1.5 cm. Each compartment was filled in with graphite granules (3–5 mm in diameter) (Xilong Chemical, China), used as the electrode material and packed enough so that the granules well contacted each other and a graphite rod (5 mm in diameter) (Xilong Chemical) to collect the electrical current. This rod penetrated the big frame of each compartment via a drilled hole (5 mm in diameter) and stuck outside. The gaps between the rod and the big frame were sealed up by epoxy glue to ensure that the compartment is closed. Also, for this purpose, rubber gaskets were placed between the poly-acrylic parts when the reactor was assembled. A 6 cm × 6 cm Nafion 117 membrane (Du Pont, USA) was used to separate the two compartments of each reactor. Each reactor was assembled using nuts and bolts penetrating holes at four corners of each big frame. Anode and cathode graphite rods were connected to crocodile clamps and through wires to an external resistor of 10 ohm and to a multimeter. Such a low resistance should allow the generation of higher current levels (Gil *et al*., 2003).

For the influent and effluent (of anolyte or catholyte), two holes (5 mm in diameter) were created on the big frame of each compartment and PVC pipes were sealed to them. The anode influent pipe was inserted with a three-way connector before connected via a drip chamber to a bottle containing modified M9 medium (0.44 g KH2PO4 l^−1^, 0.34 g K2HPO4 l^−1^, 0.5 g NaCl l^−1^, 0.2 g MgSO4.7H2O l^−1^, 0.0146 g CaCl2 l^−1^, pH 7) (Clauwaert *et al*., 2007).

The reactors were operated in batch mode at room temperature (22 ± 3°C) (unless otherwise stated). Before a batch, the M9 medium bottle was sterilized, cooled and purged with nitrogen (Messer, Vietnam) for 30–60 min to minimize the amount of oxygen, the potential competitor with the anode to accept electrons. To start a batch, a FeCl_2_ solution (the source of ferrous ions) was syringed, together with a trace element solution (with the recipe following Clauwaert *et al*., 2007), into the anode compartment of each MFC through the three-way connector on the anode influent pipe. The supplied volume and the concentration of the FeCl_2_ solution were calculated so that the final concentration of Fe^2+^ in the anolyte will be as desired. The volume of the trace element solution was also calculated so that its final proportion in the anolyte was 0.1% (v/v). Subsequently, sterilized and nitrogen-purged M9 medium was sucked from the containing bottle, with a syringe, and pumped into the anode compartment, also through the three-way connector. The volume of the pumped-in medium was calculated such that half of the anolyte was replaced (approximately 10 ml). This replacement also helped remove a part of ferric precipitate formed in the anode compartment. Finally, a NaHCO_3_ solution (the carbon source) was supplied into the anode compartment, in a similar manner, such that its final concentration in the anolyte was 2 g l^−1^ (Clauwaert *et al*., 2007). This sequence of supplying the components of the anolyte ensures that ferrous carbonate precipitate was not formed (experimentally checked, data not shown). The cathode compartment of each MFC reactor contained only a buffer solution (0.44 g KH2PO4 l^−1^, 0.34 g K2HPO4 l^−1^, 0.5 g NaCl l^−1^). At the beginning of each batch, this catholyte was renewed completely. During a batch, the cathode compartment was aerated, through the cathode influent pipe, with an air pump (model SL-2800, Silver Lake, China) to supply oxygen, the final electron acceptor. The aeration rate was adjusted to be slightly above 50 ml min^−1^ to ensure that the catholyte was air-saturated (Pham *et al*., 2005) but did not evaporate fast. A batch of operation for a reactor was timed from the moment right after the anolyte was replaced until when the current dropped down to the baseline (usually about 2 h). Each reactor was operated at least three batches per day (with 1 h being the interval between two consecutive batches) and left standby during the nighttime. (This mode of operation did not affect the stability in the performance of the reactors.)

### Measurement and calculation of electrical parameters

A digital multimeter (model DT9205A+, Honeytek, Korea) was used to measure the voltage between the anode and the cathode of each MFC. Electrical parameters [current I(A), voltage U(V) and resistance R(Ω)] were measured and/or calculated according to Aelterman and colleagues (2006) and Logan and colleagues (2006). Unless otherwise stated, all the values of average currents and charges reported in this study were the results of at least three repetitions.

### Inoculation and enrichment procedures

After assembled, all the reactors were double-checked to ensure no leakages and bad electrical connections occurring. Several MFC reactors were set up in this study. One reactor [the (biotic) control] was not initially inoculated with any microbial source but operated in the same manner as other reactors and thus could be contaminated with microbes from the environment. Furthermore, in order to prove that the generation of electricity in the MFCs was not due to plain chemical reactions, an abiotic control was used. The abiotic control was a reactor of the same MFC type, with the anode compartment (including the electrode) sterilized (at 121°C, 1 atm, for 20 min) and subsequently tested with Fe^2+^ for only 2 h right after assemblage. Three other reactors, designated as the inoculated MFCs, were inoculated with a bacterial source (an inoculum) from a natural mud taken from a brownish water stream at the depth of 20 cm underneath the stream bottom, in Ung Hoa, Hanoi, Vietnam.

The inoculation was carried out as follows: In the first 3 days, the inoculum was daily supplemented into the anode compartment of each reactor (except the control) and the reactors were operated with 20 mM of Fe^2+^. The inoculum was prepared by mixing 1 ml of sterile M9 medium with the pellet (after centrifuged at 4000× *g*, for 5 min) of 2 ml of the original bacterial source (the mud). After day 3, the reactors were operated without supplementation of inocula.

During the enrichment period (the first 4 weeks), all the MFC reactors were operated in the manner mentioned above with 20 mM of Fe^2+^ supplied into each anode compartment and the generation of electricity was monitored. Samples from their anolytes (1 ml each) were daily taken and preserved at 4°C (for later microbiological analyses) or at −20°C (for molecular analyses).

### Measurement of bacteria density and ferrous iron

The OD of bacterial cells in each anode suspension sample was measured at 600 nm using a UV/VIS spectrophotometer (BioMate 3S, Thermo Scientific, USA). Before measurement, one volume of the sample was pretreated with 1/50 volume of 25% HCl solution to prevent the formation of ferrous precipitates that might interfere the OD signal.

The concentration of Fe^2+^ in an anolyte sample was measured by the phenanthroline assay (Braunschweig *et al*., 2012). In short, 5 μl of each sample was diluted 60 times before being treated with 6 μl of 25% HCl solution as mentioned above. The resulting solution was centrifuged (4000× *g*, 10 min) and the supernatant was added with 30 μl of 5.2 M ammonium acetate solution, 12 μl of 21 mM phenanthroline solution and 255 μl of distilled water. After about 30 min, the 490 nm absorbance value of that final solution was determined using the mentioned spectrophotometer. That value was used to calculate the concentration of Fe^2+^ in the sample, based on a pre-determined calibration line.

### Isolation and morphology analyses of bacteria from the anodes of the MFCs

After the enrichment period (i.e. when the current generations of the MFCs were steady), culturable bacteria in anode suspension samples of an inoculated MFC and the (biotic) control were analysed.

Bacteria in an anode suspension sample of a MFC were plated for isolation and colony counting on Winogradsky medium (0.5 g KH2PO4 l^−1^, 0.5 g NaNO3 l^−1^, 0.2 g CaCl2 l^−1^, 0.5 g MgSO4.7H2O l^−1^, 0.5 g NH4NO3 l^−1^, 6 g Ammonium Ferric Citrat l^−1^; 16 g agar l^−1^) (Starosvetsky *et al*., 2008) by dilution method. Colonies of each isolate were counted from at least three plates per dilution level. Isolates were subcultured and preserved in Luria–Bertani (LB) medium.

Cells of isolated strains were fixed and Gram stained following standard procedures (Madigan *et al*., 2004) and observed under a light microscopy (Carl-Zeiss, Germany).

The judgment for overlapping isolates was based on colony morphology as well as cell morphology analyses.

### Molecular analyses

Preparation of samples: Anode suspension samples from the MFCs were used as such. To prepare an anode surface sample, particles on 1 cm^2^ of the electrode surface were scrapped off using a sterile razor and suspended in 1 ml of a pH 7 buffer solution (0.44 g KH2PO4 l^−1^, 0.34 g K2HPO4 l^−1^, 0.5 g NaCl l^−1^).

DNA extraction and polymerase chain reaction (PCR)-DGGE: Samples were centrifuged (4000× *g*, 10 min) and the pellets were used for DNA extraction. Total DNA of a sample was extracted using standard methods (Boon *et al*., 2000). DNA was quantified based on UV absorption at 260 nm. 16S rRNA gene fragments were amplified with the primers P63F (5′-CAGGCCTAACACATGCAAGTC-3′) and P1378R (5′-CGGTGTGTACAAGGCC CGGGAACG-3′). These fragments were used as the templates to amplify 200 bp fragments with the primers P338fGC and P518R (Muyzer *et al*., 1993). These 200 bp fragments were subjected to DGGE with a denaturing gradient ranging from 45% to 60% (Boon *et al*., 2002) to analyse the compositions of the bacterial communities in the samples. The rate of change of a community was calculated as the percentage of change in the corresponding DGGE pattern (Marzorati *et al*., 2008).

Bands of interest on DGGE gel were cut off from the gel and spliced into small pieces using a sterile razor. The small gel pieces were subsequently suspended in 50 μl of deionized water for 24 h at 4°C to allow DNA to elute. This eluted DNA was used as the template to amplify again the DNA fragment corresponding to each band. The PCR products were purified with ExoSAP – IT kit (Affymetric, USA) before submitted to Integrated ADN Technologies – IDT (Singapore) for DNA sequencing.

For 16S rDNA analysis of the isolates, each isolate was cultured in LB broth for 24 h before the cells were harvested for DNA extraction (following the same procedure). From extracted DNAs, 16S rRNA gene fragments were amplified with the primers P63F and P1378R. PCR products were similarly purified and sequenced.

The analysis of DNA sequences and homology searches were completed with standard DNA sequencing programs and the BLAST server of the National Center for Biotechnology Information using the BLAST algorithm (Altschul *et al*., 1990).

### FISH

After the enrichment, when the electrical generation was stable, samples from the anode suspension and anode surface of an inoculated MFC were taken and prepared as mentioned. Samples were fixed as follows: 1 ml of each sample was mixed with 0.1 ml of phosphate buffer saline (PBS) (10×, pH7). The mixture was supplemented with 1 ml of 37% (v/v) formaldehyde solution and kept at 4°C for at least 30 min. This mixture was subsequently centrifuged (5000× *g*, 5 min) and the pellet was washed with PBS. After washing, this cells-containing pellet was suspended in a solution containing 50% ethanol and 50% PBS solution. DNA hybridization was carried out as follows using probe PS1 (5′-ACGGUAGAGGAGCAAUC -3′) specific for neutrophilic iron-oxidizing bacteria (Siering and Ghiorse, 1997): 20 μl of each fixed sample was diluted in 2 ml of sterile deionized water and applied on a polycarbonate membrane and allow to naturally dry. Two microlitre of the probe solution (containing 5 ng μl^−1^ of PS1 bound with the fluorescent-emitting compound Cy3 and supplied by IDT) were mixed with 18 μl of hybridization buffer (including 180 μl of 5 M NaCl ml^−1^; 20 μl of 1 M Tris-HCl ml^−1^; 350 μl of formamide ml^−1^, and 1 μl of 10% SDS ml^−1^), and chilled on ice, in the dark. This mixture was dropped onto the polycarbonate membrane carrying the already dried sample. The membrane was subsequently incubated in a hybridization chamber at 46°C for 1.5–3 h. After that, this sample-carrying membrane was washed with washing buffer (including 800 μl of 5M NaCl, 1 ml of 1M Tris/HCl, 0.5 ml of 0.5M EDTA, 50 μl of 10% SDS and water in a total volume of 50 ml) and heated at 48°C for 15 min. Next, the membrane was washed in sterile water for several seconds before wiped up with sterile tissue. Each sample-carrying membrane was treated with 50 μl of 4,6-diamidino-2-phenylindole (DAPI) solution (1 μg ml^−1^) for 3 min in the dark and quickly washed with sterile water and with 80% ethanol for several seconds. Finally, after drying, the samples were observed under a fluorescent microscope (model Axiostar Plus, Carl-Zeiss, Germany) using a specialized filter (552 nm) for Cy3 signals and a blue/cyan filter (460 nm) for DAPI signals. Images of samples were captured by a camera connected to the microscope. In these images, white dots corresponding to signals from stained cells were calculated by using Image J. Thus, the quantity of white dots from DAPI signals (x) indicated the number of total bacteria while that from Cy3 signals (y) indicated the number of PS1-probed bacteria. Therefore, in each sample, the proportion (%) of iron bacteria could be calculated as y/x*100.

### Data analysis

All the experiments, unless otherwise stated, were repeated three times. Data were analysed using basic statistical methods by using Microsoft Excel: differences in data were evaluated by t-Test analysis; errors among replicates were expressed in the form of standard deviations.

## Supporting Information

**Fig. S1.** Picture of a MFC reactor in this study. The reactor was assembled from polyacrylic frames and contains two compartments separated by a Nafion 117 membrane. Graphite granules were used as the electrode matrixes connecting with graphite rods at both compartments. Red wires connect an external circuit with the cathode while black wires connect it with the anode. Plastic tubes are for the influent and the effluent of purged air at the cathode and for the influent and the effluent of ‘fuel’ solution at the anode. The device is operated with an external resistor of 10 ohm at 25°C. **Fig. S2.** Per-batch coulombic amounts generated by the MFCs in this study. Each coulombic amount (Q) is calculated as: Q = I × t; whereas I is the current intensity (A) and t is the average time of a batch (from the feeding moment till the moment the current decreases by 95% of the maximum steady current). **Appendix S1. DNA sequences in this study**
